# Neurosurgical leadership in neuro-oncology clinical trials: A nationwide study

**DOI:** 10.1007/s10143-026-04165-5

**Published:** 2026-03-09

**Authors:** Christian K. Ramsoomair, Manav Daftari, Akhil Desai, Victoria Alvarez, Vratko Himic, Manuela Aramburu Berckemeyer, Nathan A. Shlobin, Adham M. Khalafallah, Sarah Wang, Bradley Elder, Gavin P. Dunn, Timothy R. Smith, Michael E. Ivan, Ricardo J. Komotar, Ashish H. Shah

**Affiliations:** 1 Department of Neurosurgery, University of Miami Miller School of Medicine, Miami, FL USA; 2 Section of Virology and Immunotherapy, Department of Neurosurgery, Leonard M. Miller School of Medicine, University of Miami, Miami, FL 33136 USA; 3 Department of Neurosurgery, Neurological Institute of New York, Columbia University Irving Medical Center, New York, NY USA; 4 Department of Neurosurgery, The Ohio State University, Columbus, OH USA; 5 Department of Neurosurgery, Massachusetts General Hospital, Boston, MA USA; 6 Department of Neurosurgery, Brigham and Women’s Hospital, Boston, MA USA; 7 Medical Scientist Training Program, University of Miami, Miller School of Medicine, Miami, FL USA

**Keywords:** Neurosurgery, Clinical trials, Principal investigator, Central nervous system (CNS) tumor, Neuro-oncology

## Abstract

Despite therapeutic advances, central nervous system (CNS) tumors such as glioblastoma and brain metastases remain highly lethal. Neurosurgeons have historically driven innovation in neuro-oncology but may be underrepresented as principal investigators (PIs) in clinical research. We analyzed all 525 U.S.-based neuro-oncology trials listed on ClinicalTrials.gov as of October 2024 to evaluate trends in neurosurgeon-led research, stratified by phase, funding source, and institutional characteristics. Oncologists led 34.8% of trials, whereas neurosurgeons led 21.5%. When normalized to the number of board-certified specialists, neurosurgeons demonstrated greater per-capita leadership than oncologists. Neurosurgeon-led studies were primarily early-phase (36.7% phase I; 5.1% phase III/IV), underscoring their role in translational and first-in-human investigations. At the state level, neurosurgical leadership correlated strongly with the number of neurosurgical residency programs (R² = 0.4835, p < 0.001) and aggregated departmental NIH funding (R² = 0.6146, p < 0.001), with states possessing greater training infrastructure and research resources demonstrating higher rates of neurosurgeon-led clinical trials. Nevertheless, 55.3% of neurosurgeon PIs lacked direct NIH support, highlighting persistent funding barriers. Notably, women were nearly twice as likely as men (1.8×) to serve as PIs, suggesting emerging gender diversity in neurosurgical research leadership. Neurosurgeons maintain a significant presence in neuro-oncology clinical trials, particularly in early translational work. However, their limited representation in NIH-sponsored and late-phase studies reflects enduring structural and funding challenges. Strengthening research mentorship, expanding grant access, and fostering interdisciplinary collaboration may enhance neurosurgical leadership and accelerate therapeutic innovation in neuro-oncology.

**Supplementary Information:**

The online version contains supplementary material available at 10.1007/s10143-026-04165-5.

## Introduction

Despite significant global progress in cancer treatment, tumors of the central nervous system (CNS), such as glioblastoma (GBM) and metastatic brain disease, continue to have dismal prognoses with ineffective therapies. GBM, the most common primary malignant brain tumor, remains universally fatal with an overall survival of less than two years, even with intensive multimodal therapy. The complex nature of these tumors, coupled with the blood-brain barrier (BBB), which limits drug delivery, necessitates innovative approaches to therapeutic development.

The neuro-oncology landscape is shaped by multidisciplinary collaboration and is rapidly evolving. Historically, neurosurgeons have primarily focused on operative management and clinical care, contributing significantly to the development of innovative surgical tools and techniques. In recent years, however, they have also emerged as key leaders in translational research and clinical trial design. Several landmark neurosurgeon-led trials underscore the importance and impact of surgical leadership in neuro-oncology, including the Phase I trial of intratumoral delivery of CAN-3110, an oncolytic herpes simplex virus (NCT03152318); the Phase III trial of the dendritic cell vaccine DCVax-L (NCT00045968); and the Phase I trial of CARv3-TEAM-E T cells targeting both EGFRvIII and wild-type EGFR (NCT05660369) [1–3].

The role of a principal investigator (PI) or senior investigator in clinical trials is to lead the study design, secure funding, oversee patient enrollment, ensure compliance with regulatory requirements, and manage data collection and analysis. Uniquely qualified for this responsibility, neurosurgeons possess a combination of technical expertise, clinical insight, and longitudinal patient access that is critical for advancing therapeutic strategies. Neurosurgeons are involved at every stage of disease management, from initial diagnosis and tumor resection to post-operative care and long-term disease monitoring. Furthermore, neurosurgeons’ direct access to tumor tissue has facilitated the seamless integration of translational research components, such as tumor antigen discovery and molecular profiling, into clinical trials, effectively bridging the gap between laboratory discoveries and patient care. 

Despite these advantages, neurosurgeons have recently been reported to be underrepresented as principal investigators in neuro-oncology clinical trials. In 2021, a study reported a declining relative rate of procedural GBM trials over time [4]. Moreover, even within this subset, neurosurgeons served as PIs or co-PIs in only about 55% of cases. While this trend is undoubtedly multifactorial and may partly reflect the evolving role neurosurgical innovation, it raises concerns about diminishing neurosurgeon leadership in clinical research. In response, Vogelbaum and colleagues identified key areas for improvement within organized neurosurgery, including enhancing educational opportunities during training and improving positioning to secure external funding [5]. 

This study aims to evaluate current trends in neuro-oncology clinical research to assess neurosurgeon involvement as principal investigators. By analyzing active and upcoming clinical trials, we hope to provide an up-to-date view on neurosurgical footprint in clinical trials, identify areas where leadership can be expanded, and propose strategies to strengthen neurosurgeons’ roles in trial leadership.

## Methods

### Search strategy and exclusion criteria

The ClinicalTrials.gov database was searched for all current and future neuro-oncology clinical trials as of October 2024, capturing all trials available prior. The following search criteria: “Brain Tumor OR Glioblastoma OR High-Grade Glioma OR Glioma OR Diffuse Intrinsic Pontine Glioma OR Diffuse Midline Glioma” All trials included in this study had “Recruiting” or “Not Yet Recruiting” status. Each clinical trial was analyzed by two independent reviewers. Clinical trials were excluded from analysis if trials did not include neurologic malignancies or trials were located outside of the United States (US). Trials involving overlapping conditions were identified and indexed according to ClinicalTrials.gov classification and were not double counted. Both adult and pediatric neuro-oncology trials were included in the analysis.

### Datapoint extraction

Trial characteristics including phase, conditions, interventions, location, sponsor and collaborators, study locations, and dates were extracted. Trials were first annotated to identify the clinical specialty of the study principal investigator (PI). Principal investigator specialty was determined using publicly available institutional profiles and published literature. Specialty assignment reflected the investigator’s final clinical specialty rather than formal residency training. Then, we re-analyzed the trials to assess whether they were *neurosurgeon-led trials*, which we assigned if neurosurgeons served as the study PI or as senior investigators at one of the clinical trial locations. If neurosurgeons participated as a study PI or senior investigator, demographic analysis was performed for each neurosurgeon, including the sex of the PI, the state where the neurosurgeon is leading the clinical trial, and funding allocated by the NIH for each PI. Neurosurgeon-led clinical trials were excluded from analysis if they were located outside of the US. Geographic distribution of neurological surgery residency program was extracted from the AAMC ERAS Directory (https://systems.aamc.org/eras/erasstats/par/index.cfm). Lastly, each neurosurgeon-led clinical trial was analyzed based on its primary intervention type and categorized into four distinct categories: (1) Drug, (2) Procedure, (3) Combination of Drug and Procedure, and (4) Device.

### Normalization methods for specialty populations and gender

To account for differences in workforce size across specialties, we normalized the number of principal investigators (PIs) by dividing the raw PI count in each specialty by the 10-year total of new specialty certificates issued physicians in that field, as reported by the American Board of Medical Specialties (ABMS). The following estimates were used: neurosurgery, 1865; medical oncology, 6,001; diagnostic radiology, 14,987; radiation oncology, 1884; neurology, 3113; pediatrics, 15,546; and internal medicine, 63,605. We termed the resulting values as Investigator-to-Workforce Ratio (IWR), highlighting the proportion of investigators relative to specialty size. For gender-specific analyses within neurosurgery, we applied an estimated 6.8% female representation among board-certified neurosurgeons, based on publicly available data from the American Board of Neurological Surgery (ABNS) and national workforce reports [6–8]. Assuming that 6.8% of neurosurgeons nationally are female, we estimated the expected number of female principal investigators (PIs) in our sample by multiplying the total number of neurosurgeon PIs by 0.068. We then compared the observed proportion of female neurosurgeons in our dataset to this expected value to assess whether female representation in our sample was higher or lower than anticipated based on national demographics.

### NIH funding metrics

Data regarding NIH funding for either principal investigators or neurosurgery departments was extracted from datasets distributed by the Blue Ridge Institute for Medical Research (BRIMR) as compiled by Robert Roskoski Jr. and Tristram G. Parslow (https://brimr.org/brimr-rankings-of-nih-funding-in-2023/). All data was derived from NIH year-end composite data for the federal fiscal year ending 30 September 2023, as released on the NIH Research Portfolio Online Reporting Tool. Correlational analysis was performed to analyze geographical correlation between NIH funding and neurological surgery residency programs. To confirm NIH funding amounts for each respective neurosurgeon PI, two independent reviewers verified amounts as documented on NIH RePORT Expenditures and Results (RePORTER). Amounts were verified by cross-referencing to the BRIMR dataset.

### Figure creation and statistical Analysis

Statistical analysis was performed using GraphPad Prism version 10.4 for MacOS (GraphPad Software, Boston, Massachusetts USA). Figures were generated using R, utilizing packages such as ggplot2, dplyr, tidyr, maps, and readxl. Correlational studies were performed using regression analysis, with significance defined as *p* < 0.05.

## Results

### Trial characteristics

**Fig. 1:**
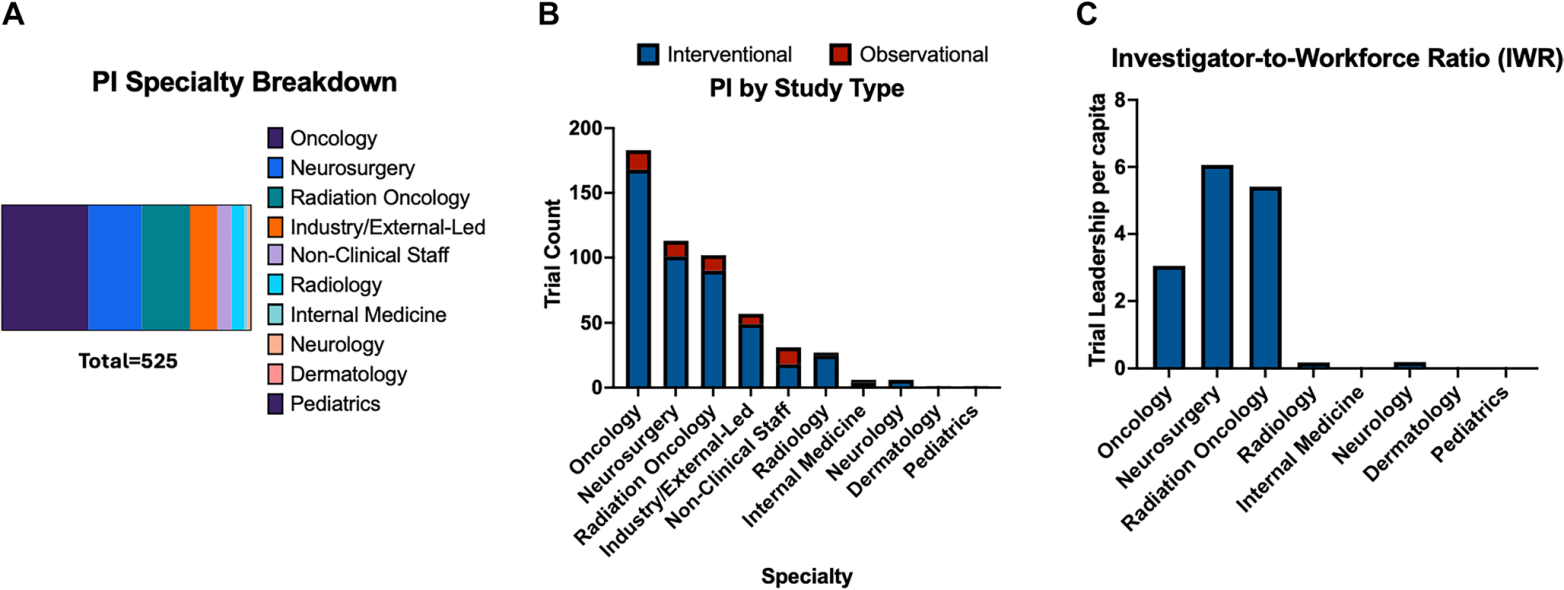
Leadership by specialty in future neuro-oncology clinical trials in USA . (**A**) Distribution of PI specialties in upcoming neuro-oncology clinical trials (**B**) Study type quantification by PI specialty (**C**) Distribution of PI specialties normalized for workforce population in upcoming neuro-oncology clinical trials

A total of 617 clinical trials were identified through ClinicalTrials.gov, of which 525 satisfied all inclusion criteria and none of the exclusion criteria. Among these, oncologists, including neuro-oncologists and medical oncologists, led 183 trials (34.8%), the highest proportion. Medical oncologists and neuro-oncologists served as study PIs for 27 (14.7%) and 157 (85.3%) trials, respectively. Further analysis examined the training backgrounds of the neuro-oncologists leading these trials. Of the 157 trials, neuro-oncologists trained in internal medicine led 42 trials (26.8%), those trained in neurology led 58 trials (36.9%), and those trained in pediatrics led 54 trials (34.4%). Neurosurgeons served as the study PI or senior investigator in 117 trials (21.5%), followed by radiation oncologists with 102 trials (19.4%). Of the 117 trials, neurosurgeons served as the study PI in 96.4% of these trials. In four clinical trials, neurosurgeons served as senior investigators with study PI specialties including neuro-oncology, radiation oncology, and non-clinical staff. Other specialties included were industry/externally led groups, non-clinical staff, radiology, internal medicine, neurology, dermatology, and pediatrics (Fig. 1A). Of note, 2 clinical trials had more than one Study PI with different clinical specialties, not including neurosurgery. In these cases, study PIs were counted twice in analysis. 

Each trial was further classified as observational or interventional. Of the total clinical trials included in the primary analysis, 463 were interventional and 64 were observational. Mirroring the overall pattern of clinical trials, oncologists and neurosurgeons primarily led interventional trials, 168 (36.3%) and 101 (21.7%), respectively. Radiation oncologists led 9 interventional trials, the third most (19.4%). Oncologists led 15 observational studies (23.4%), followed by neurosurgeons, 12, (18.8%). Radiation oncologists led 12 observational trials, the third most (18.8%). (Fig. 1B).

Using specialty population data obtained through the ABMS, we derived the Investigator-to-Workforce Ratio (IWR) as the proportion of principal investigators relative to specialty size. Neurosurgery had the highest IWR (6.1), followed by radiation oncology (5.4) and oncology (3.0) (Fig. 1C). Other specialties such as internal medicine, pediatrics, and radiology were less common in neuro-oncology clinical trial leadership.

### Phase distribution of neurosurgeon-led trials

**Fig. 2:**
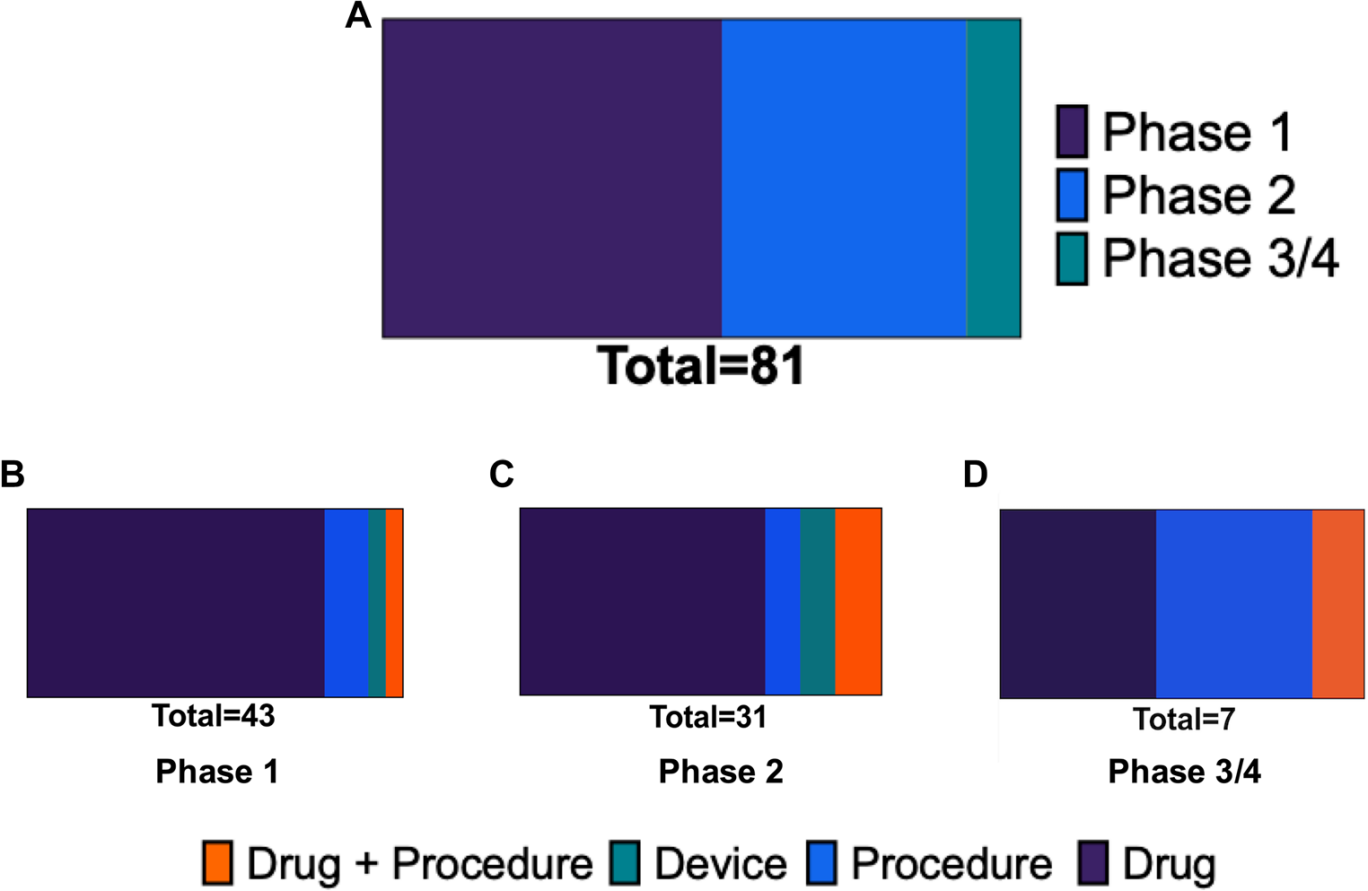
Distribution and biases in neurosurgeon-led clinical trials by phase and intervention type. (**A**) Overall distribution of neurosurgeon-led clinical trials by trial phase. (B–D) Intervention type breakdown within each trial phase: phase 1 (**B**), phase 2 (**C**), and phase 3/4 (**D**). Intervention types include drug, device, procedure, behavioral, biological, and other categories. * Excluding undesignated phases

In total, 117 trials involved neurosurgeons serving as the study PI or a senior investigator. When stratified by phase, forty-three neurosurgeon-led clinical trials are currently in phase 1 (36.7%) while only 31 are phase 3 studies (5.1%), and only a single trial is currently in phase 4 (0.8%) (Fig. 2A). Further trial characteristics, including study status, interventions, start/completion dates, sponsors, and collaborators, are reported in the following tables: Supplementary Table 1 presents observational trial characteristics for studies without a designated phase. Supplementary Tables 2, 3, and 4 summarize clinical trials in Phases 1, 2, and 3/4, respectively.

### Primary intervention types of neurosurgeon-led trials

Across all phases, most neurosurgeon-led clinical trials involved the investigation of a novel or repurposed pharmacologic treatment for CNS malignancies. In phase 1 studies, over 75% of studies involved investigation of drugs followed by procedural investigations (12.9%) (Fig. 2B). A similar trend was observed in phase 2 studies with 67.7% for studies investigating drugs, 9.7% for procedural investigations, and 12.9% for drug and procedural investigations. (Fig. 2C) However, in phase 3 and 4 studies, there was an enrichment on novel procedures for treating CNS malignancies (42.9%) (Fig. 2D).

### Geographic distribution

**Fig. 3:**
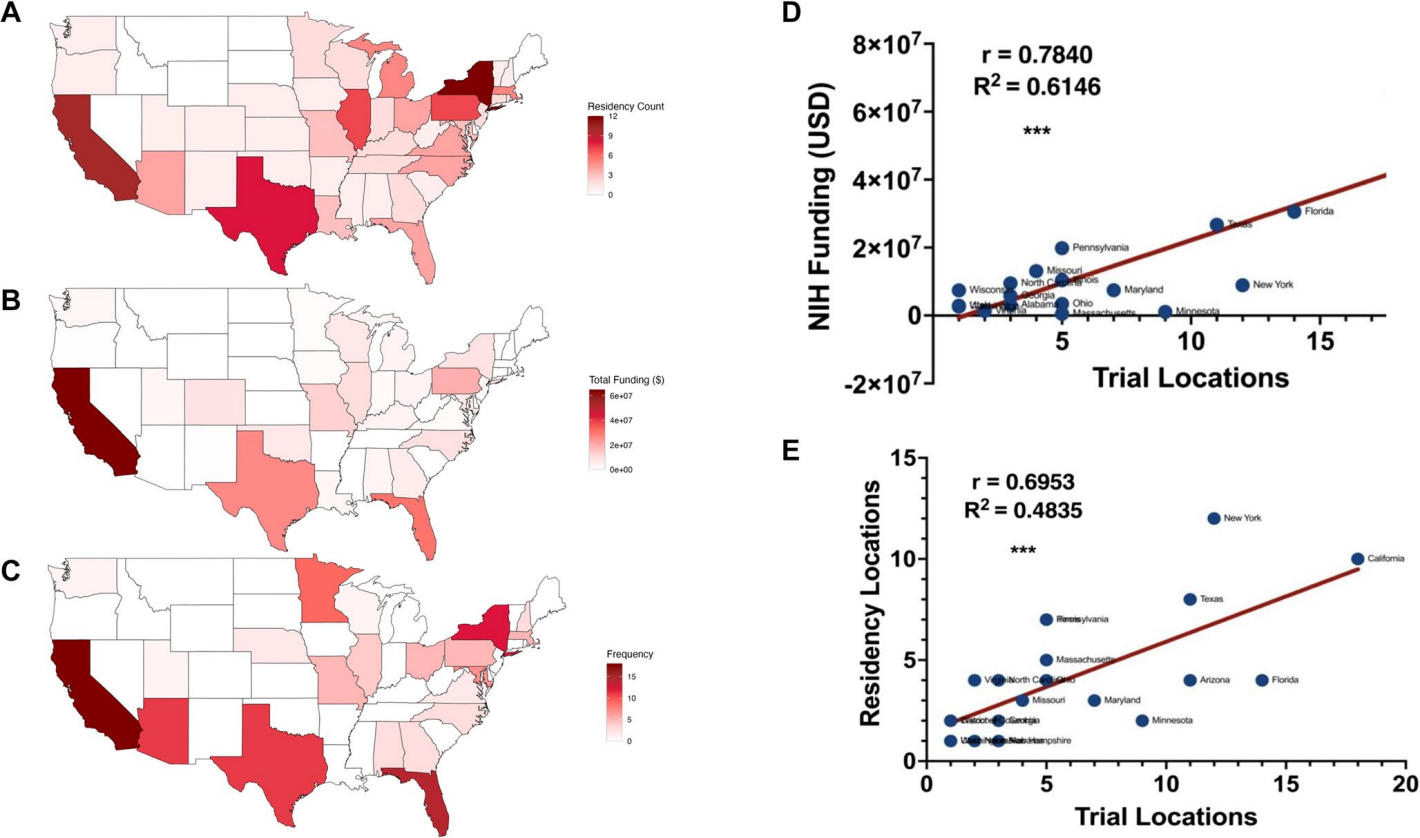
Geographic mapping shows correlation between number of neurosurgeon-led trials, academic medical centers, and departmental NIH funding. (**A**) Neurological surgery residency programs, (**B**) NIH funding of neurosurgery departments, and (**C**) Neurosurgeon-led clinical trial frequency by state. (**D**) Correlation between NIH funding of neurosurgery departments and neurosurgeon-led clinical trial frequency. (**E**) Correlation between number of residency programs and neurosurgeon-led clinical trial frequency

In addition to gaining a better understanding of the geographical distribution of trials across the U.S., we used state-level data as a framework to examine potential associations among three key variables: neurosurgical leadership, NIH funding, and the number of academic neurosurgery departments. By aggregating and analyzing these data at the state level, we were able to compare trends across regions and conduct linear regression analyses to explore potential correlations and underlying patterns. We report a breakdown of states with the most trials (Fig. 3A). A majority of trials are located in California (14.3%), Florida (11.1%), New York (9.5%), Texas (8.7%), Arizona (8.7%), and Minnesota (7.1%). Figure 3B and C illustrate a similar geographic distribution between neurosurgeon-led clinical trials and neurosurgical residency programs or NIH neurosurgery departmental funding respectively. We observed a strong correlation between distribution of NIH funding for neurosurgical residency programs and neurosurgeon-led clinical trials (R^2^ = 0.6146, *p* < 0.001) (Fig. 3D). Additionally, we found a moderate correlation between distribution of neurosurgeon-led clinical trials and neurosurgical residency programs (R^2^ = 0.4835, *p* < 0.001) (Fig. 3E). Notably, California, Texas, and Florida consistently lead the USA with neurosurgical academic programs, NIH neurosurgery departmental funding, and neurosurgeon-led clinical trials.

### Gender distribution of neurosurgeon clinical trial PIs

As of October 2024, nine female neurosurgeons (12.0%) were study PIs or senior investigators in neurooncological clinical trials that are recruiting or not yet recruiting. After normalizing for gender proportions in the neurosurgical workforce, women neurosurgeons were found to be approximately 1.8x times more likely than males to serve as PIs (Supplemental Fig. 1). We also sought to determine if the number of registered clinical trials associated with an individual neurosurgeons differed by the identified gender of the male and female neurosurgeon. There was no statistical difference between male and female neurosurgeons with regards to their number of registered clinical trials (data not shown).

### NIH funding assessment for neurosurgeon trial PIs

**Fig. 4:**
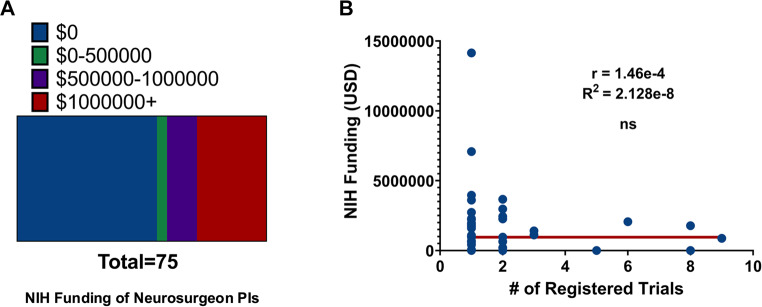
Majority of Neurosurgeon Trial PIs receive no NIH funding. (**A**) Funding distribution of NIH funding among Neurosurgeon-trial PIs categorized by total funding amounts, (**B**) Linear regression analysis between NIH funding and the number of registered clinical trials led by individual neurosurgeon investigators demonstrates no significant correlation (R² = ~0, not significant)

Last, we sought to better understand what the impact of federal (NIH) funding was on neurosurgeon-involvement in clinical trials. For observational, phase 1, phase 2, and phase 3 + trials, the NIH was found to be a sponsor on only 3 (8.3%), 0 (0%), 2(%), and 0 (0%) respectively, and a collaborator on 8 (22.2%), 8 (18.6%), 6 (19.3%), and 0 (0%) respectively. Strikingly, sourcing data from NIH RePORTER and the BRIMR dataset, we found that 55.3% of neurosurgeons in upcoming neuro-oncologic clinical trials receive $0 in NIH funding as a principal investigator (Fig. 4A). Additionally, no correlation was found between the amount of NIH funding each neurosurgeon receives and the number of current-led clinical trials (Fig. 4B).

## Discussion

Neurosurgeons play a fundamental role in the field of neuro-oncology, contributing to diagnosis, treatment, and translational research. However, recent reports suggest underrepresentation of neurosurgeons as principal investigators in neuro-oncology clinical trials. Neurosurgeons have historically played a pivotal role in both establishing early study groups (i.e. Brain Tumor Study Group and Brain Tumor Cooperative Group) and advancing surgical techniques and acquiring specimens for brain tumor management. Our findings indicate that neurosurgeons play a vital and sustained leadership role in the field.

### Current landscape of neurosurgeons in neuro-oncology clinical trials

The gradual diminution of neurosurgeon leaders in neuro-oncology clinical trials has been noted previously, primarily regarding declines in procedure-centric trials [4]. While the raw data show that neurosurgeons lead nearly a 1/3 less of total interventional trials than that of the top specialty, oncology, this information may be misleading if not put in the proper context. Given the relatively small size of the neurosurgical workforce, we used the Investigator-to-Workforce Ratio (IWR) to compare leadership between specialties. Representing the proportion of investigators relative to specialty size, we found that neurosurgery had the highest IWR. Our analysis revealed that neurosurgeons are predominantly involved in early-phase trials, with 36.7% of neurosurgeon-led studies classified as phase 1 and 5.1% classified as phase 3/4. While neurosurgeons appear to be maintaining a strong presence in trial leadership, there may be opportunities to expand their involvement in later-phase trials. Current barriers for neurosurgeons could include securing funding, establishing multi-institutional collaborations, or designing large-scale studies that integrate real-time biospecimen acquisition and other surgical advancements. Moreover, the inherent complexity of surgery-centered trials poses additional obstacles. Unlike drug trials, which can often be standardized as “drug versus placebo,” surgical investigations must contend with heterogeneity in patient anatomy, operative techniques, instrumentation, and surgeon experience. Ensuring consistent methodology across surgeons and institutions is therefore particularly difficult, further complicating the conduct of late-phase neurosurgical trials.

With regards to gender, we found that females are approximately 1.8 times more likely to serve as PIs. This contributes to a growing body of literature emphasizing the importance of the increasing presence and impact of female surgeons in surgical specialties, particularly in neurosurgery [9, 10]. Our findings emphasize the importance of continued support for groups such as Women in Neurosurgery (WINS), which has driven increases in female neurosurgical residents from 13% in 2010 to 24% in 2022 [11]. It is important to note that estimates of female investigator representation were extrapolated from published neurosurgical workforce data, and thus, further research is encouraged to directly ascertain gender-based differences in neurosurgical study PIs.

### Evolving role of neurosurgeons in neuro-oncology

While the principal investigator role of neurosurgeons in neuro-oncology trials has traditionally been procedure-centric, our data shows that neurosurgeons maintain a strong presence in leadership despite relative declines in procedure-centric trials over time. Neurosurgical innovations address one of the greatest challenges in neuro-oncology: the delivery of systemic treatments to CNS tumors, particularly high-grade gliomas. Techniques such as Ommaya reservoir placement and convection-enhanced delivery (CED) for targeted drug delivery have revolutionized treatment strategies, reducing toxicity while improving therapeutic penetration [12]. Moreover, the neurosurgical operating room provides a unique environment for translational research, particularly in Phase 0 studies and Window-of-opportunity trials, which assess drug penetration into tumor tissue and help bridge basic science with clinical application or allow for early proof-of-concept testing, expediting drug delivery and biodistribution assessments, respectively [5, 13]. As such, we encourage current neurosurgeon-led observational trials to progress into interventional studies, particularly in early-phase trials assessing the safety, feasibility, and efficacy of intraoperative modalities such as laser interstitial thermal therapy (LITT), intraoperative brachytherapy, photodynamic therapy (PDT), sonodynamic therapy (SDT), and focused ultrasound (FUS). Additionally, we have observed a clear evolution in neurosurgery toward novel, multimodal therapeutic design, such as CAR T cells, oncolytic viruses, and other immunomodulatory agents delivered locoregionally, positioning neurosurgeon-scientists at the forefront of these innovations [1–3]. 

### Investing in neurosurgeon-led neuro-oncology trials

**Fig. 5:**
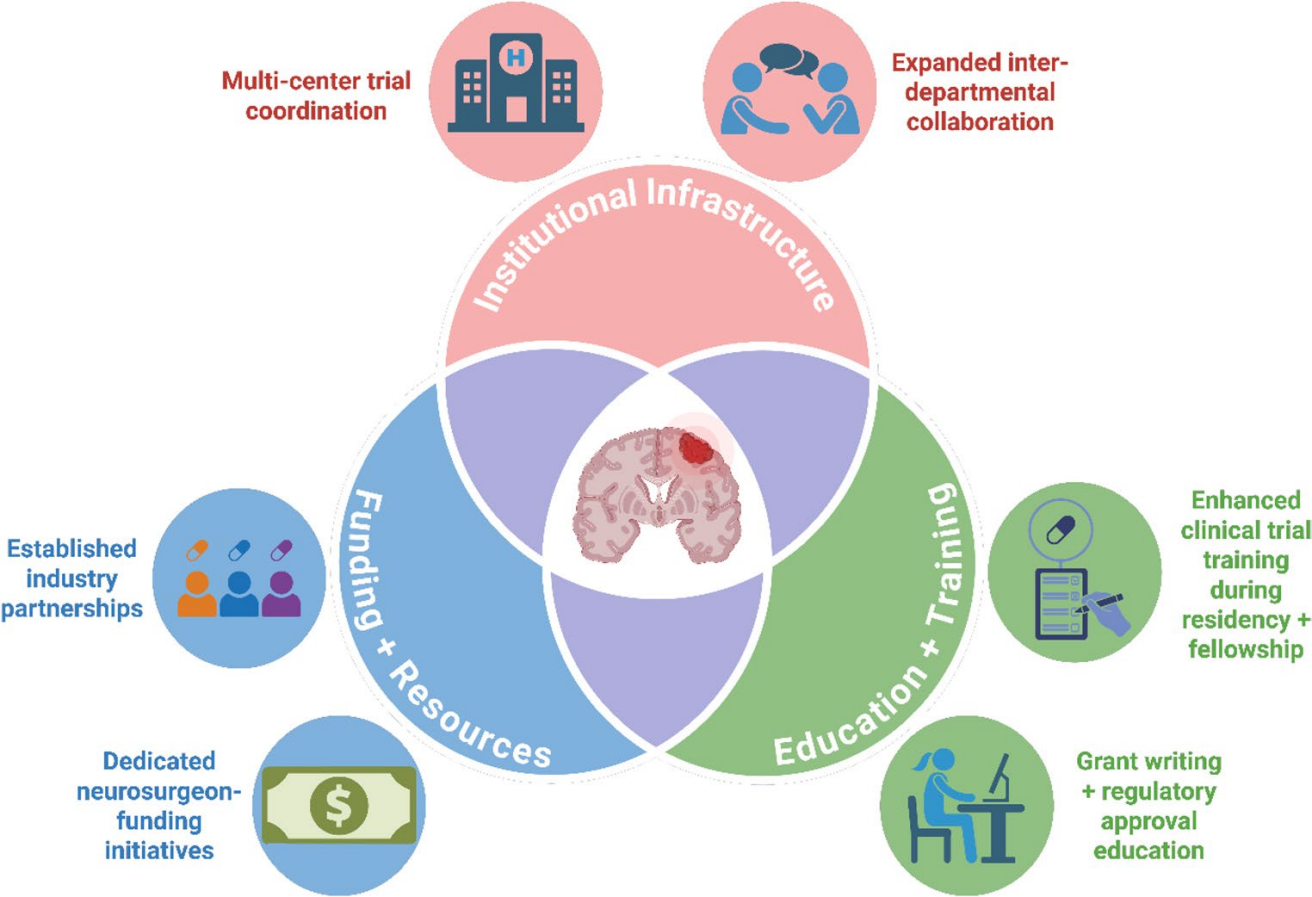
Strategic pathways for enhancing neurosurgeon leadership in clinical trials. This figure presents a framework for expanding neurosurgeon leadership/involvement in clinical trials by focusing on three core areas: institutional infrastructure, funding and resources, and education and training. Institutional challenges include limited collaboration across departments and a lack of support for coordinating multi-center trials. On the funding side, obstacles such as minimal industry partnerships and few neurosurgeon-specific funding programs can limit research participation. Education remains a major gap, with few opportunities during residency or fellowship to gain experience in trial design, regulatory processes, or grant development. Addressing these issues together may help overcome key deterrents and create a more supportive environment for neurosurgeon-led clinical research. Created in Biorender.com

While our study suggests that neurosurgeon leadership in neuro-oncology clinical trials is strong, there are opportunities for growth, particularly in later-phase trials. Efforts to increase overall neurosurgeon leadership will undoubtedly be multifactorial (Fig. 5). Deterrents currently include financial disincentives (research endeavors vs. cases), lack of formal training in trial design, and limited research funding opportunities [5, 14]. During neurosurgery residency, there is often limited exposure to the conception, regulatory planning, and execution of clinical trials, particularly those involving drug development or multi-arm randomized protocols. Medical oncologists, on the other hand, typically train in environments where clinical trials are core to practice. Furthermore, there are no established, paid fellowships in neurosurgery focused on clinical trial design or clinical research infrastructure training, in contrast to other specialties where such opportunities are common. Another significant barrier to neurosurgeon-led clinical trials is the lack of connectivity with the pharmaceutical and biotechnology sectors, which often serve as the primary engines behind modern therapeutic development. Unlike in oncology or radiology, where industry relationships are more firmly established, such partnerships remain underdeveloped in neurosurgery and thus limit opportunities. At the institutional level, neurosurgeons are frequently evaluated and rewarded based on operative volume and clinical productivity, with little protected time or financial support allocated for research activities. This creates a disincentive to engage in the resource-intensive process of initiating and leading clinical trials.

Our geographic distribution analysis showed that neurosurgeon-led trials were most concentrated in states with strong academic centers and NIH-funded departments such as California, Florida, New York, Texas, Arizona, and Minnesota. Thus, this highlights that institutional support, access to collaborative networks, and localized training opportunities are critical determinants of trial leadership. Support for clinical trial development in neurosurgery could be strengthened by leveraging established programs such as the Neurosurgeon Research Career Development Program (NRCDP K12), the Neurosurgery Research & Education Foundation (NREF), and the NIH Neurosurgeon-Scientist Training (NST) pipeline [14]. Dedicated initiatives like NREF continue to play a pivotal role in supporting pilot projects that can lead to larger, extramurally funded studies. Notably, every $1 invested by NREF in residents and early-career neurosurgeons has generated $36 in subsequent NIH funding [15]. However, areas for growth still remain as our analysis revealed that fewer than 5% of neurosurgeon-led clinical trials are supported by the NIH, and the majority of neurosurgeon PIs (55.3%) receive no NIH funding annually (Fig. 4A).

The value of neurosurgeons in clinical trial leadership does not stem from exclusivity of expertise but from the uniquely strategic vantage point they occupy at the intersection of diagnosis, intervention, and tissue access. Neurosurgeons serve as the gateway for many patients entering neuro-oncology care. They are often the first specialist to see in the patient after hospital admission, the first to obtain definitive pathological tissue, and the primary decision-makers in surgical eligibility and risk stratification. This position enables them to influence not just treatment plans, but also research design and biospecimen availability. Previous work by the EORTC Brain Tumor Group has highlighted a lack of standardization in surgical trial methodologies and challenges in randomization due to equipoise concerns [16]. Efforts to improve trial design like establishing surgical quality indicators and standardized terminology are needed to ensure that neurosurgical advancements translate effectively into clinical practice [17]. For instance, trials assessing novel local therapies or intraoperative imaging and navigation technologies are inherently dependent on surgical logistics, workflow, and feasibility. Without neurosurgeon engagement at the study design stage, these trials risk being misaligned with the practical constraints of the operating room or underutilizing opportunities for intraoperative data acquisition. Moreover, neurosurgeons are uniquely positioned to facilitate longitudinal tumor sampling, an essential component of modern translational research. Access to matched pre-treatment, post-treatment, and recurrent tumor tissue is critical for understanding treatment resistance, tumor evolution, and clonal dynamics. Also, sequentially obtained tissue may be utilized for vaccine development and drug level assessments [18]. Furthermore, neurosurgeon-led or co-led trials may help bridge important cultural and disciplinary divides that still exist within neuro-oncology. For example, integrating surgical endpoints into medical oncology-driven trials can foster a more holistic approach to patient care and enable trials to ask broader, more patient-centered questions, such as how therapeutic sequencing or the timing of surgery affects immune response, neurocognitive outcomes, or functional independence [19–21]. 

### Limitations

While our study provides valuable insights into the landscape of neurosurgeon-led neuro-oncology clinical trials, several limitations must be acknowledged. First, our analysis relied on data extracted from only trials with “Recruiting” or “Not Yet Recruiting” status via ClinicalTrials.gov, which limits this study to only trials that are currently enrolling patients or plan to enroll in the future. Our exclusion of international trials limits the generalizability of our findings beyond the U.S., though it allows for a focused examination of neurosurgeon involvement in domestic research.

Another limitation is with the classification of principal investigators by specialty. While every effort was made to accurately identify neurosurgeon-led trials, inconsistencies in trial reporting or multi-specialty leadership structures may have influenced our results. Investigator roles and specialties were derived from publicly reported trial records and external institutional sources, which may not reflect changes in leadership over the course of a study or informal leadership contributions not formally listed. Additionally, ClinicalTrials.gov reporting practices vary across sponsors and institutions, potentially introducing misclassifications of leadership roles.

While we attempted to directly compare PIs from medical neuro-oncology and surgical neuro-oncology, disparate training paths and lack of up-to-date available demographics made this not possible. Federal funding disparities also represent a potential limitation in our study that warrants further exploration. While our study highlights a lack of NIH funding among neurosurgeons leading clinical trials, other funding sources such as private foundations, industry sponsorship, and institutional support were not assessed. Future studies should analyze the impact of alternative funding mechanisms on neurosurgeon-led research and evaluate how financial incentives or institutional policies influence trial leadership.

### Conclusion and future directions

Sustaining and expanding neurosurgical leadership in clinical trials will require coordinated investment in early training, incentive structures, and equitable access. Dedicated support for early-career neurosurgeon–scientists is paramount, and should include protected research time, starter and bridge funding, and structured cross-departmental mentorship. Institutional reward systems should explicitly acknowledge the value of trial conception, leadership, high-quality patient enrollment, and regulatory oversight in promotion and compensation decisions, rather than viewing these activities as secondary to revenue generation. Equally important is early, substantive exposure during training: residency and fellowship curricula should include formal instruction in trial methodology and biostatistics, rotations with clinical trial offices, and co–principal-investigator apprenticeships that normalize clinical investigation as a core professional pathway.

Expanding neurosurgeon-led trials beyond a select number of highly resourced centers is essential to prevent further concentration of trial leadership. Building capacity at emerging sites through mentored onboarding, hub-and-spoke consortia, and the use of central Institutional Review Boards (IRBs) can reduce start-up barriers and create more equitable opportunities. Trial methods and infrastructure should also be tailored to the realities of neurosurgery, incorporating pragmatic and registry-based randomized designs, expertise-based randomization, standardized operative reporting, and integrated biospecimen collection. Progress should be monitored with transparent, field-wide metrics, such as the number of neurosurgeon-led phase II/III trials, the geographic diversity of participating sites, and the extent of trainee involvement in leadership roles. Collectively, these measures can help promote neurosurgical innovation translates into practice-changing evidence and, ultimately, improved patient outcomes.

## Supplementary Information

Below is the link to the electronic supplementary material.

Supplementary figure 1 Female leadership in clinical trials appears to outpace their presence in the neurosurgeon workforce. (**A**) Gender distribution of neurosurgeon PIs in upcoming neuro-oncology clinical trials. (**B**) Gender distribution in the neurosurgeon workforce. (PNG 462 KB).

High Resolution Image (TIFF) 2.16 MB

Supplementary Material 2 (DOCX 28.0 KB)

Supplementary Material 3 (DOCX 34.6 KB)

Supplementary Material 4 (DOCX 31.4 KB)

Supplementary Material 5 (DOCX 24.8 KB)

## Data Availability

All data supporting the findings of this study are publicly available from ClinicalTrials.gov, National Institutes of Health (NIH) RePORTER, and Blue Ridge Institute for Medical Research databases. The datasets analyzed during the current study are available from these sources without restriction. Derived data supporting the results of this analysis are available from the corresponding author upon reasonable request.
