# Supplementary figures and images for: Neurosurgical leadership in neuro-oncology clinical trials: A nationwide study
Source: Neurosurg Rev. 2026 Mar 9;49(1):265. doi: 10.1007/s10143-026-04165-5 (PMC12971842; doi:10.1007/s10143-026-04165-5)

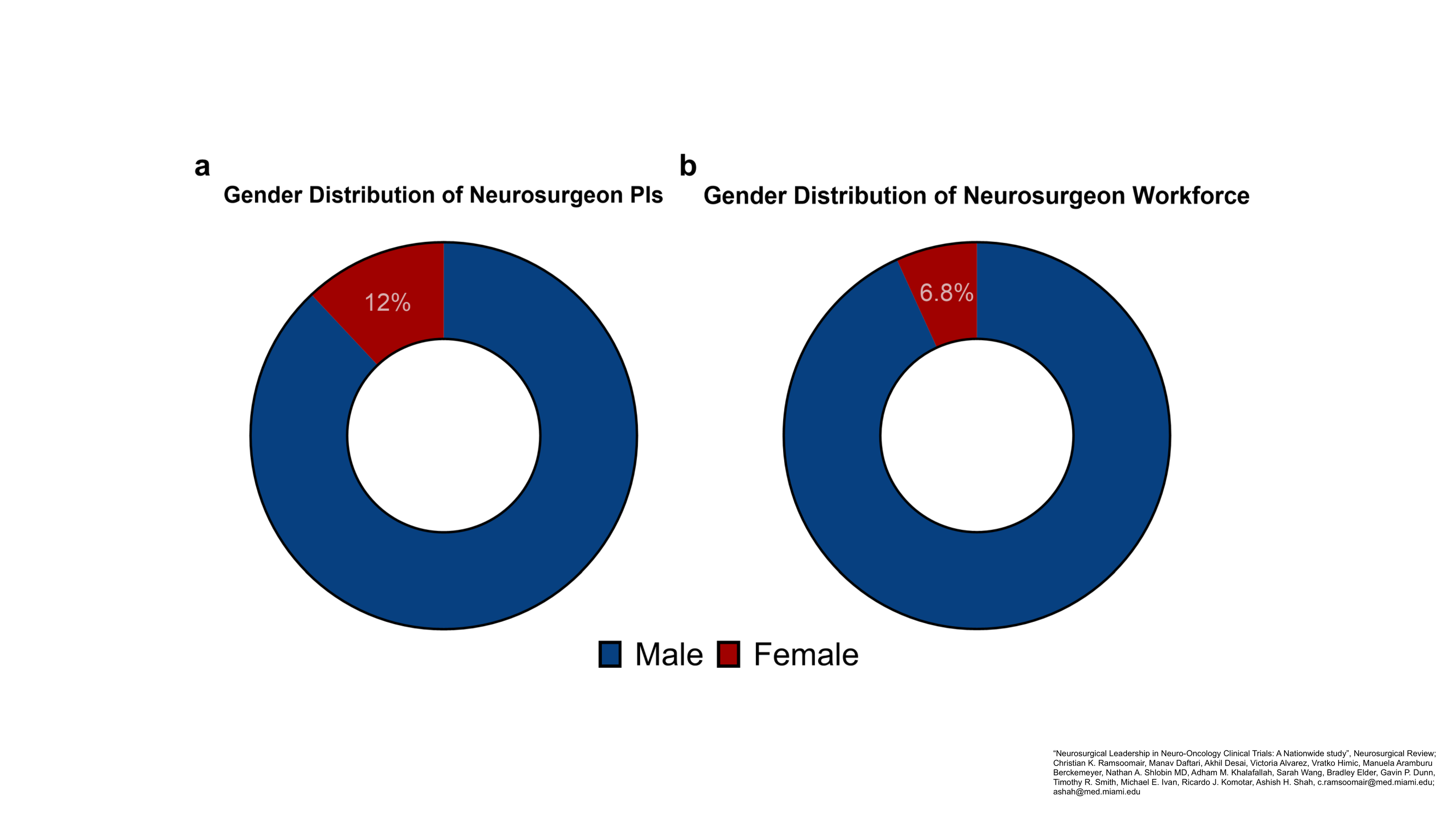

Supplement: Supplementary file 1 — Female leadership in clinical trials appears to outpace their presence in the neurosurgeon workforce. (A) Gender distribution of neurosurgeon PIs in upcoming neuro-oncology clinical trials. (B) Gender distribution in the neurosurgeon workforce. (PNG 462 KB). [file 10143_2026_4165_Fig6_ESM.png]

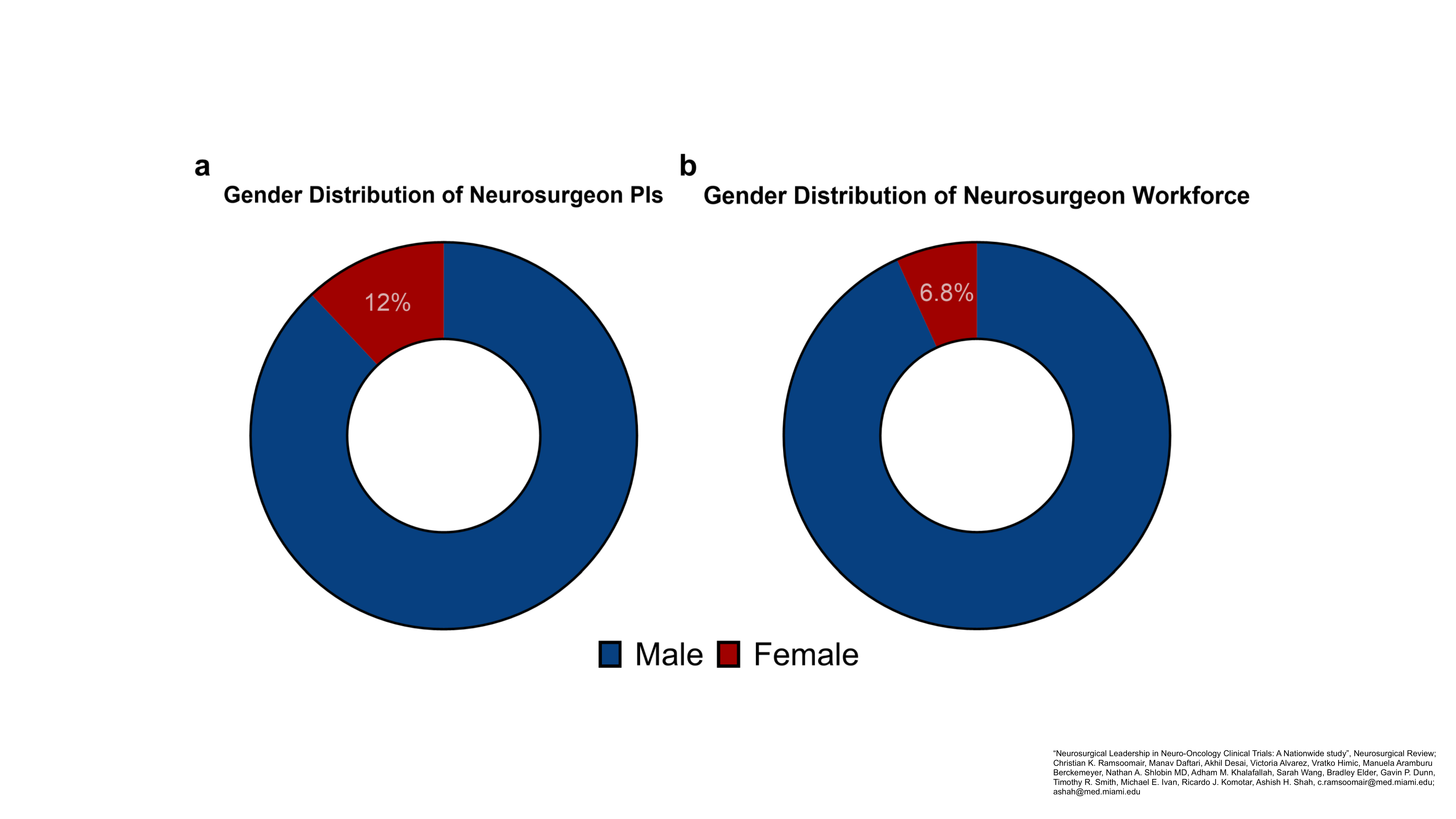

Supplement: Supplementary file 2 — High Resolution Image (TIFF) 2.16 MB [file 10143_2026_4165_MOESM1_ESM.tiff]
