# Supplementary material for: Neurosurgical leadership in neuro-oncology clinical trials: A nationwide study
Source: Neurosurg Rev. 2026 Mar 9;49(1):265. doi: 10.1007/s10143-026-04165-5 (PMC12971842; doi:10.1007/s10143-026-04165-5)
Supplement: Supplementary file 3 — Supplementary Material 2 (DOCX 28.0 KB) [file 10143_2026_4165_MOESM2_ESM.docx]

| **NCT Number** | **Study Title** | **Study Status** | **Interventions** | **Start Date** | **Completion Date** | **Sponsor** | **Collaborators** |
| --- | --- | --- | --- | --- | --- | --- | --- |
| NCT05979064 | Omental Tissue Autograft In Human Recurrent Glioblastoma Multiforme (Rgbm) | Recruiting | Laparoscopically Harvested Omental Tissue Autograft | 04/2023 | 04/2027 | Northwell Health |  |
| NCT05954858 | Surgical Tissue Flap To Bypass The Blood Brain Barrier In Glioblastoma | Recruiting | Tissue Autograft Of Pedicled Temporoparietal Fascial (Tpf) Or Pericranial Flap To Bypass The Blood Brain Barrier (Bbb) | 06/2023 | 06/2027 | Northwell Health |  |
| NCT05139277 | Evaluation Of The Convivo System | Recruiting | Convivo System \| Conventional Histologic Evaluation | 06/2022 | 06/2025 | Dartmouth-Hitchcock Medical Center | Carl Zeiss Meditec, Inc. |
| NCT03677999 | Spectroscopic Magnetic Resonance Imaging Of Glioma | Recruiting | Mega-Press Sequence Magnetic Resonance Spectroscopy | 06/2019 | 09/2025 | University of Minnesota |  |
| NCT06387979 | Advanced Development Of Desorption Electrospray Ionization Mass Spectrometry For Intraoperative Molecular Diagnosis Of Brain Cancer Using Pathology Biopsies | Recruiting | Non-Interventional Study | 10/2020 | 10/2025 | Mayo Clinic | National Cancer Institute (NCI) |
| NCT05513859 | Investigational Imaging Technique During Brain Surgery | Not Yet Recruiting | Craniotomy \| Quantitative Oblique Back-Illumination Microscopy | 10/2024 | 06/2025 | Emory University | National Cancer Institute (NCI) |
| NCT01849952* | Evaluating The Expression Levels Of Microrna-10B In Patients With Gliomas | Recruiting |  | 10/2013 | 05/2025 | Dartmouth-Hitchcock Medical Center |  |
| NCT04810871 | Surgical Resection Of Latent Brain Tumors Prior To Recurrence | Recruiting | Brain Surgery | 05/2021 | 03/2026 | Mayo Clinic |  |
| NCT06630338 | Use Of Shear Wave Elastography For Intraoperative Brain And Glioma Stiffness Measurements | Not Yet Recruiting | Non-Interventional Study | 02/2025 | 02/2033 | Mayo Clinic |  |
| NCT03542409 | Safety And Feasibility Of Preoperative And Intraoperative Image-Guided Resection Of Gliomas | Recruiting | Mr Perfusion Scan \| 2Hg Spectroscopy Scan \| Standard Of Care Intraoperative Mri | 02/2017 | 07/2026 | University of Utah |  |
| NCT06322602 | Ommaya Reservoir Placement At The Time Of Biopsy For Longitudinal Biomarker Collection In Patients With Brain Tumors | Recruiting | Intracranial Catheter Placement \| Lumbar Puncture \| Biopsy \| Biospecimen Collection \| Computed Tomography | 02/2024 | 03/2029 | Mayo Clinic |  |
| NCT00811148 | Florida Center For Brain Tumor Research | Recruiting | Tissue Bank | 03/2006 | 12/2025 | University of Florida |  |
| NCT04692337 | Ommaya Reservoir Placement For Brain Tumor Biomarker Access | Recruiting | Intra-Operative Ommaya Reservoir Placement | 01/2021 | 12/2025 | Mayo Clinic |  |
| NCT02754544 | Electrocorticography In Mapping Functional Brain Areas During Surgery In Patients With Brain Tumors | Recruiting | Direct Electrocortical Stimulation \| Electrocorticography | 07/2016 | 01/2028 | M.D. Anderson Cancer Center | National Cancer Institute (NCI)\|University of Houston |
| NCT05733312 | Extracellular Impact Of Ultrasound-Induced Blood-Brain Barrier Disruption | Recruiting | Insightec'S Exablate Neuro Model 4000 Type 2.0 (220 Khz) System | 01/2024 | 01/2025 | Mayo Clinic |  |
| NCT05789862 | Behavioral Health Evaluation And Intervention Program For Patients Undergoing Craniotomy | Not Yet Recruiting | Counseling Sessions \| Quality Of Life Questionnaires | 08/2024 | 12/2025 | University of California, San Francisco | Clarity Squared Behavioral, Inc |
| NCT01535430 | Assessment Of Eloquent Function In Brain Tumor Patients | Recruiting | Brain Mapping | 01/2012 | 12/2025 | University of Nebraska |  |
| NCT04742231 | Handheld Dynamometer During Awake Craniotomy Pilot | Recruiting | Hand-Held Dynamometer | 07/2020 | 12/2024 | Mayo Clinic |  |
| NCT04047264 | Feasibility Of Intraoperative Microdialysis During Neurosurgery For Central Nervous System Malignancies | Recruiting | Microdialysis | 01/2020 | 09/2027 | Mayo Clinic | National Cancer Institute (NCI)\|National Institute of Neurological Disorders and Stroke (NINDS) |
| NCT02639325 | Tumor Related Epilepsy | Recruiting |  | 01/2016 | 07/2032 | National Institute of Neurological Disorders and Stroke (NINDS) |  |
| NCT05281731 | Sonobiopsy For Noninvasive And Sensitive Detection Of Glioblastoma | Recruiting | Sonobiopsy \| Research Blood \| Cancer Personalized Profiling \| Definity¬¨√Ü | 04/2022 | 04/2025 | Washington University School of Medicine | National Cancer Institute (NCI) |
| NCT06176066 | Ph Sensitive Mri Based Resections Of Glioblastoma | Not Yet Recruiting | Cest Ph Mri Based Resection Of Glioblastoma | 01/2024 | 01/2029 | University of California, Los Angeles |  |
| NCT04657146 | Longitudinal Assessment Of Marrow And Blood In Patients With Glioblastoma | Recruiting | Biorepository | 02/2024 | 11/2026 | Duke University |  |
| NCT05864976 | Neurosurgical Neuronavigation Using Resting State Mri And Machine Learning | Recruiting | Support Vector Machine | 12/2023 | 01/2030 | Washington University School of Medicine | National Cancer Institute (NCI) |
| NCT05989893 | The Neural Code And Dynamics Of The Reading Network. | Recruiting | Language-Based Tasks | 08/2022 | 07/2027 | The University of Texas Health Science Center, Houston | National Institute of Neurological Disorders and Stroke (NINDS) |
| NCT04463979 | Perioperative Evaluation Of Cerebellar Tumors | Recruiting | Impact Of Cerebellar Functional Topography On Cognition And Motor Ataxia | 02/2021 | 04/2026 | Duke University |  |
| NCT04692324 | Cerebrospinal Fluid Biomarkers For Brain Tumors | Recruiting | Biospecimen Collection \| Electronic Health Record Review \| Survey Administration | 01/2021 | 12/2025 | Mayo Clinic | National Cancer Institute (NCI)\|National Institute of Neurological Disorders and Stroke (NINDS) |
| NCT06117930 | Study Of Human Brain-Gut Axis And Gut Microbiome In Patients With Brain Lesions - Repository For Neuroscience Research | Recruiting | Non-Interventional Study | 02/2024 | 12/2029 | Mayo Clinic |  |
| NCT04822688 | Analysis Of Cell Count, Viability, And Immunogenicity Of Discarded Newly Diagnosed Glioblastoma Tissue Or Solid Tumor Tissues | Recruiting | Biospecimen Collection | 02/2021 | 12/2024 | Thomas Jefferson University |  |
| NCT03028246 | A Feasibility Safety Study Of Benign Centrally-Located Intracranial Tumors In Pediatric And Young Adult Subjects | Recruiting | Exablate 4000 System | 02/2017 | 12/2024 | InSightec |  |
| NCT05755399 | Feasibility Of Transcranial Focused Ultrasound To Measure Brain Tumor | Recruiting | Brain Imaging Using Transcranial Focused Ultrasound (Tfus) | 10/2023 | 09/2026 | University of Minnesota |  |
| NCT05124912 | Remaster: Recurrent Brain Metastases After Srs Trial | Recruiting | Radiation Therapy \| Steroid Therapy \| Laser Interstitial Thermal Therapy | 05/2022 | 10/2028 | Monteris Medical |  |
| NCT05659524 | Nasal Outcomes Using Saline Irrigations After Endonasal Pituitary Surgery | Recruiting | Nasal Saline Irrigation | 12/2020 | 12/2029 | Lori Wood | Barrow Brain and Spine |
| NCT03678389 | Feasibility Of Endosphenoidal Coil Placement For Imaging Of The Sella During Transsphenoidal Surgery | Recruiting | Esc | 05/2019 | 12/2028 | National Institute of Neurological Disorders and Stroke (NINDS) |  |
| NCT04087902 | Long-Term Longitudinal Qol In Patients Undergoing Eea | Recruiting |  | 07/2019 | 12/2025 | Ohio State University |  |
| NCT04569591 | Corticotrophin-Releasing Hormone (Crh) Stimulation For 18F-Fdg-Pet Detection Of Pituitary Adenoma In Cushing S Disease | Not Yet Recruiting | Acthrel | 10/2024 | 12/2025 | National Institute of Neurological Disorders and Stroke (NINDS) |  |

Supplementary Table 1. Neurosurgeon-led undesignated phase trials.
