# Supplementary material for: Neurosurgical leadership in neuro-oncology clinical trials: A nationwide study
Source: Neurosurg Rev. 2026 Mar 9;49(1):265. doi: 10.1007/s10143-026-04165-5 (PMC12971842; doi:10.1007/s10143-026-04165-5)
Supplement: Supplementary file 4 — Supplementary Material 3 (DOCX 34.6 KB) [file 10143_2026_4165_MOESM3_ESM.docx]

| **NCT Number** | **Study Title** | **Phases** | **Study Status** | **Interventions** | **Start Date** | **Completion Date** | **Sponsor** | **Collaborators** |
| --- | --- | --- | --- | --- | --- | --- | --- | --- |
| NCT05773326 | Superselective Intra-arterial Cerebral Infusion of Temsirolimus in HGG | Early Phase1 | Recruiting | Temsirolimus | 05/2023 | 04/2026 | Nader Sanai | Barrow Neurological Institute\|Ivy Brain Tumor Center |
| NCT06072586 | Study of BDTX-1535 in Recurrent High-Grade Glioma (HGG) Participants With EGFR Alterations or Fusions | Early Phase1 | Recruiting | BDTX-1535 | 10/2023 | 10/2025 | St. Joseph's Hospital and Medical Center, Phoenix | Ivy Brain Tumor Center\|Barrow Neurological Institute |
| NCT04559685 | Study of Sonodynamic Therapy in Participants With Recurrent High-Grade Glioma | Early Phase1 | Recruiting | SONALA-001(ALA) and MR-Guided Focused Ultrasound device (MRgFUS) \| MR-Guided Focused Ultrasound device (MRgFUS) | 03/2021 | 03/2025 | Nader Sanai | Barrow Neurological Institute\|Ivy Brain Tumor Center\|SonALAsense, Inc.\|InSightec |
| NCT05717153 | Intratumoral Extracellular Metabolic Impact of DFMO and AMXT 1501 in Patients With Diffuse or High Grade Glioma | Early Phase1 | Recruiting | Biospecimen Collection \| Computed Tomography \| Eflornithine \| Magnetic Resonance Imaging \| Polyamine Transport Inhibitor AMXT-1501 Dicaprate \| Resection \| Microdialysis \| Placement | 10/2023 | 09/2027 | Mayo Clinic | National Cancer Institute (NCI) |
| NCT05076513 | Trial of Niraparib in Participants With Newly-diagnosed Glioblastoma and Recurrent Glioma | Early Phase1 | Recruiting | Niraparib \| Radiation therapy | 10/2021 | 02/2025 | Nader Sanai | Barrow Neurological Institute\|Ivy Brain Tumor Center\|University of California, San Francisco\|GlaxoSmithKline |
| NCT04869449 | Neuro-pharmacological Properties of Repurposed Ketoconazole in Glioblastomas | Early Phase1 | Recruiting | Ketoconazole | 05/2022 | 01/2025 | Milton S. Hershey Medical Center |  |
| NCT04825275 | Neuro-pharmacological Properties of Repurposed Posaconazole in Glioblastoma: A Phase 0 Clinical Trial | Early Phase1 | Recruiting | Posaconazole Pill | 02/2022 | 01/2025 | Milton S. Hershey Medical Center | National Cancer Institute (NCI) |
| NCT05182905 | AZD1390 in Recurrent and Newly Diagnosed WHO Grade 4 Glioma Patients | Early Phase1 | Recruiting | AZD1390 | 03/2022 | 01/2025 | Nader Sanai | Barrow Neurological Institute\|Ivy Brain Tumor Center\|AstraZeneca |
| NCT04135807 | Implantable Microdevice In Primary Brain Tumors | Early Phase1 | Recruiting | Microdevice | 03/2020 | 02/2024 | Oliver Jonas, PhD |  |
| NCT04391595 | LY3214996 Plus Abemaciclib in Recurrent Glioblastoma Patients | Early Phase1 | Recruiting | Abemaciclib \| LY3214996 | 07/2020 | 02/2025 | Nader Sanai | Barrow Neurological Institute\|Ivy Brain Tumor Center\|Eli Lilly and Company |
| NCT06636162 | Window of Opportunity Study of DSP-0390 in Gliomas | Early Phase1 | Not Yet Recruiting | DSP-0390 | 12/2024 | 02/2026 | Washington University School of Medicine | Sumitomo Pharmaceuticals America |
| NCT02905110 | Methotrexate and Etoposide Infusions Into the Fourth Ventricle in Children With Recurrent Posterior Fossa Brain Tumors | Early Phase1 | Recruiting | Methotrexate \| Etoposide \| Ommaya Reservoir | 10/2016 | 11/2025 | The University of Texas Health Science Center, Houston |  |
| NCT05798507 | Identification of Treatment Concentrations of Defactinib or VS-6766 for the Treatment of Patients With Glioblastoma | Early Phase1 | Recruiting | Avutometinib \| Biospecimen Collection \| Defactinib | 07/2023 | 10/2026 | Emory University | National Cancer Institute (NCI)\|Verastem, Inc. |
| NCT04614909 | Study of Pamiparib in Newly Diagnosed and rGBM | Early Phase1 | Recruiting | Pamiparib \| Olaparib \| Radiation therapy \| Temozolomide | 01/2021 | 06/2025 | Nader Sanai | Barrow Neurological Institute\|Ivy Brain Tumor Center\|BeiGene |
| NCT05303467 | A Feasibility Study to Evaluate the Safety of the TheraSphere Glioblastoma (GBM) Device in Patients With Recurrent GBM | Early Phase1 | Recruiting | TheraSphere GBM | 07/2022 | 06/2025 | Boston Scientific Corporation |  |
| NCT05380349 | Personalized Cancer Stem Cell High-Throughput Drug Screening for Glioblastoma | Early Phase1 | Recruiting | Combinations of up to 3 FDA approved drugs from a panel of compounds | 04/2024 | 12/2027 | Swedish Medical Center |  |
| NCT04315064 | Infusion of Panobinostat (MTX110) Into the Fourth Ventricle in Children and Adults With Recurrent Medulloblastoma | Early Phase1 | Recruiting | MTX110 | 04/2020 | 12/2025 | The University of Texas Health Science Center, Houston | Midatech Pharma plc |
| NCT04958486 | Combination Intraventricular Chemotherapy Pilot Study: 5-Azacytidine (5-AZA) and Trastuzumab Infusions Into the Fourth Ventricle or Resection Cavity in Children and Adults With Recurrent or Residual Posterior Fossa Ependymoma | Early Phase1 | Recruiting | 5-Azacytidine and trastuzumab infusion | 07/2021 | 12/2025 | The University of Texas Health Science Center, Houston |  |
| NCT04837547 | PEACH TRIAL- Precision Medicine and Adoptive Cellular Therapy | Phase 1 | Recruiting | Tumor-specific ex vivo expanded autologous lymphocyte transfer (TTRNA-xALT) | 09/2021 | 09/2030 | University of Florida | Beat Childhood Cancer Research Consortium |
| NCT06428045 | STARLITE for Unresectable High-Grade Gliomas | Phase 1 | Not Yet Recruiting | Magnetic Resonance (MR)-guided Laser Interstitial Thermal Therapy (LITT) \| Abacavir \| Lamivudine \| Ritonavir \| Temozolomide \| Focal Radiotherapy | 11/2024 | 05/2029 | University of Miami | Medtronic |
| NCT03011671 | Study of Acetazolamide With Temozolomide in Adults With Newly Diagnosed or Recurrent Malignant Glioma | Phase 1 | Recruiting | Acetazolamide \| Temozolomide | 10/2018 | 10/2026 | University of Chicago |  |
| NCT06463184 | Study to Assess Xevinapant in Preoperative Subjects With Recurrent High-Grade Glioma (rHGG) | Phase 1 | Recruiting | Xevinapant | 07/2024 | 06/2027 | H. Lee Moffitt Cancer Center and Research Institute | EMD Serono Research & Development Institute, Inc. |
| NCT05660369 | CARv3-TEAM-E T Cells in Glioblastoma | Phase 1 | Recruiting | CARv3-TEAM-E T cells | 03/2023 | 06/2026 | Marcela V. Maus, M.D.,Ph.D. |  |
| NCT05478837 | Genetically Modified Cells (KIND T Cells) for the Treatment of HLA-A*0201-Positive Patients With H3.3K27M-Mutated Glioma | Phase 1 | Recruiting | Cyclophosphamide \| Fludarabine \| Autologous Anti-H3.3K27M TCR-expressing T-cells | 07/2023 | 08/2029 | University of California, San Francisco | The V Foundation\|Parker Institute for Cancer Immunotherapy\|Alliance for Cancer Gene Therapy |
| NCT06193174 | Re-Administration of C134 in Patients With Recurrent GBM (C134-HSV-1) | Phase 1 | Not Yet Recruiting | C134 Re-Administration | 08/2025 | 08/2027 | University of Alabama at Birmingham |  |
| NCT03152318 | A Study of the Treatment of Recurrent Malignant Glioma With rQNestin34.5v.2 | Phase 1 | Recruiting | rQNestin \| Cyclophosphamide \| Stereotactic biopsy | 07/2017 | 06/2026 | Dana-Farber Cancer Institute | National Institutes of Health (NIH)\|Candel Therapeutics, Inc. |
| NCT06614855 | A Phase IB 2 Dose Trial of IRS-1 HSV C134 (IND 17296) Administered Intratumorally in Patients With Recurrent Malignant Glioma | Phase 1 | Not Yet Recruiting | C134 | 01/2025 | 01/2027 | James Markert, MD |  |
| NCT03896568 | MSC-DNX-2401 in Treating Patients With Recurrent High-Grade Glioma | Phase 1 | Recruiting | Oncolytic Adenovirus Ad5-DNX-2401 \| Therapeutic Conventional Surgery | 02/2019 | 09/2027 | M.D. Anderson Cancer Center | DNAtrix, Inc. |
| NCT06455189 | Magnetic Resonance Fingerprinting Guided Extended Resection in Glioblastomas | Phase 1 | Not Yet Recruiting | Control Group - Standard of care neurosurgical resection \| MRF/MRI infiltration guidance for extended resection | 12/2024 | 12/2029 | Case Comprehensive Cancer Center |  |
| NCT05139056 | Multiple Doses of Neural Stem Cell Virotherapy (NSC-CRAd-S-pk7) for the Treatment of Recurrent High-Grade Gliomas | Phase 1 | Recruiting | Neural Stem Cells-expressing CRAd-S-pk7 \| Resection | 05/2023 | 08/2025 | City of Hope Medical Center | National Cancer Institute (NCI) |
| NCT05363826 | Intracavitary Photodynamic Therapy as an Adjuvant to Resection of Glioblastoma or Gliosarcoma Using IV Photobac¬Æ | Phase 1 | Recruiting | Photochemotherapy using 3-(1-Butyloxy)ethyl-3-deacetyl-bacteriopurpurin-18-n-butylimide methyl ester(Photobac¬Æ) | 04/2023 | 05/2026 | Photolitec LLC | Roswell Park Cancer Institute\|National Cancer Institute (NCI) |
| NCT04214392 | Chimeric Antigen Receptor (CAR) T Cells With a Chlorotoxin Tumor-Targeting Domain for the Treatment of MMP2+ Recurrent or Progressive Glioblastoma | Phase 1 | Recruiting | Chlorotoxin (EQ)-CD28-CD3zeta-CD19t-expressing CAR T-lymphocytes (via ICT delivery) \| Chlorotoxin (EQ)-CD28-CD3zeta-CD19t-expressing CAR T-lymphocytes (via ICT/ICV dual delivery) | 02/2020 | 08/2025 | City of Hope Medical Center | National Cancer Institute (NCI) |
| NCT04661384 | Brain Tumor-Specific Immune Cells (IL13Ralpha2-CAR T Cells) for the Treatment of Leptomeningeal Glioblastoma, Ependymoma, or Medulloblastoma | Phase 1 | Recruiting | IL13Ralpha2-specific Hinge-optimized 41BB-co-stimulatory CAR Truncated CD19-expressing Autologous T-Lymphocytes | 03/2021 | 11/2025 | City of Hope Medical Center | National Cancer Institute (NCI) |
| NCT06039709 | Sonodynamic Therapy in Patients With Recurrent GBM | Phase 1 | Recruiting | 5-ALA and Low-Intensity Focused Ultrasound (SDT) | 01/2024 | 06/2026 | Shayan Moosa, MD |  |
| NCT02285959 | Super-Selective Intraarterial Intracranial Infusion of Bevacizumab (Avastin) for Glioblastoma Multiforme | Phase 1 | Recruiting | Bevacizumab | 06/2014 | 06/2025 | Global Neurosciences Institute |  |
| NCT04003649 | IL13Ra2-CAR T Cells with or Without Nivolumab and Ipilimumab in Treating Patients with GBM | Phase 1 | Recruiting | IL13Ralpha2-specific Hinge-optimized 4-1BB-co-stimulatory CAR/Truncated CD19-expressing Autologous TN/MEM Cells \| Ipilimumab \| Nivolumab \| Quality-of-Life Assessment \| Questionnaire Administration | 12/2019 | 03/2025 | City of Hope Medical Center |  |
| NCT05720624 | Pharmacodynamic Analyses of Metabolic Agents Following Brain Radiation | Phase 1 | Not Yet Recruiting | Anhydrous Enol-oxaloacetate \| Best Practice \| Biospecimen Collection \| Magnetic Resonance Spectroscopic Imaging \| Questionnaire Administration | 03/2025 | 12/2027 | Mayo Clinic |  |
| NCT06342908 | A Vaccine (Neoantigen-Targeted ppDC) for the Treatment of H3 G34-mutant Diffuse Hemispheric Glioma | Phase 1 | Not Yet Recruiting | Biospecimen Collection \| Dendritic Cell Therapy \| Leukapheresis \| Magnetic Resonance Imaging \| Poly ICLC | 12/2024 | 01/2028 | Jonsson Comprehensive Cancer Center |  |
| NCT05457959 | Peptide-Pulsed Dendritic Cell Vaccination in Combination With Nivolumab and Ipilimumab for the Treatment of Recurrent and/or Progressive Diffuse Hemispheric Glioma, H3 G34-mutant | Phase 1 | Not Yet Recruiting | Dendritic Cell Tumor Peptide Vaccine \| Ipilimumab \| Leukapheresis \| Nivolumab \| Placebo Administration \| Placebo Administration \| Poly ICLC \| Resection | 12/2024 | 05/2030 | Jonsson Comprehensive Cancer Center |  |
| NCT05789394 | Allogenic Adipose-Derived Mesenchymal Stem Cells for the Treatment of Recurrent Glioblastoma or Recurrent Astrocytoma in Patients Undergoing Craniotomy | Phase 1 | Recruiting | Allogeneic Adipose-derived Mesenchymal Stem Cells \| Biospecimen Collection \| Craniotomy \| Magnetic Resonance Imaging \| Ommaya Reservoir Tap | 06/2023 | 07/2025 | Mayo Clinic |  |
| NCT04994977 | Intra-Arterial Chemotherapy for Newly Diagnosed, Residual, or Recurrent Atypical Choroid Plexus Papilloma and Choroid Plexus Carcinoma Prior to Second-Look Surgery | Phase 1 | Recruiting | Melphalan \| Carboplatin \| Topotecan | 05/2023 | 12/2026 | Weill Medical College of Cornell University |  |
| NCT03572530 | Infusion of 5-Azacytidine (5-AZA) Into the Fourth Ventricle in Patients With Recurrent Posterior Fossa Ependymoma | Phase 1 | Recruiting | 5-Azacytidine (5-AZA) Dose Dependent on Groups | 02/2019 | 12/2025 | The University of Texas Health Science Center, Houston |  |
| NCT06466798 | Fourth Ventricular Administration of Immune Checkpoint Inhibitor (Nivolumab) and Methotrexate or 5-Azacytidine for Recurrent Medulloblastoma, Ependymoma, and Other CNS Malignancies | Phase 1 | Recruiting | Nivolumab \| Methotrexate \| 5-Azacytidine | 07/2024 | 07/2027 | David Ilan Sandberg |  |

Supplementary Table 2. Neurosurgeon-led phase 1 trials.
