# Supplementary material for: Neurosurgical leadership in neuro-oncology clinical trials: A nationwide study
Source: Neurosurg Rev. 2026 Mar 9;49(1):265. doi: 10.1007/s10143-026-04165-5 (PMC12971842; doi:10.1007/s10143-026-04165-5)
Supplement: Supplementary file 5 — Supplementary Material 4 (DOCX 31.4 KB) [file 10143_2026_4165_MOESM4_ESM.docx]

| **NCT Number** | **Study Title** | **Phases** | **Study Status** | **Interventions** | **Start Date** | **Completion Date** | **Sponsor** | **Collaborators** |
| --- | --- | --- | --- | --- | --- | --- | --- | --- |
| NCT05956821 | Treatment of Relapsed/Refractory Intracranial Glioma in Patients Under 22 Years of Age | Phase 1 \|Phase 2 | Recruiting | SIACI of cetuximab and bevacizumab | 12/2024 | 12/2029 | University of Miami |  |
| NCT03510208 | Panitumumab-IRDye800 in Diagnosing Participants With Malignant Glioma Undergoing Surgery | Phase 1 \|Phase 2 | Recruiting | Near-Infrared Fluorescence Imaging \| Panitumumab \| Panitumumab-IRDye800 \| POINPOINT-IR9000 | 05/2018 | 12/2024 | Eben Rosenthal | National Institutes of Health (NIH) |
| NCT03213002 | Oral Capecitabine and Temozolomide (CAPTEM) for Newly Diagnosed GBM | Phase 1 \|Phase 2 | Recruiting | Capecitabine \| Temozolomide | 06/2017 | 06/2026 | Northwell Health |  |
| NCT02861898 | Super-selective Intra-arterial Repeated Infusion of Cetuximab for the Treatment of Newly Diagnosed Glioblastoma | Phase 1 \|Phase 2 | Recruiting | Intra-arterial Cetuximab \| Intra-arterial Mannitol | 06/2016 | 12/2026 | Northwell Health |  |
| NCT04528680 | Ultrasound-based Blood-brain Barrier Opening and Albumin-bound Paclitaxel and Carboplatin for Recurrent Glioblastoma | Phase 1 \|Phase 2 | Recruiting | Sonication for opening of blood-brain barrier \| Chemotherapy, albumin-bound paclitaxel \| Chemotherapy, carboplatin | 10/2020 | 09/2025 | Northwestern University | CarThera\|Bristol-Myers Squibb\|Lantheus Medical Imaging |
| NCT04977375 | Trial of Anti-PD-1 Immunotherapy and Stereotactic Radiation in Patients With Recurrent Glioblastoma | Phase 1 \|Phase 2 | Recruiting | Pembrolizumab \| Stereotactic Radiation Therapy \| Surgical Resection | 12/2021 | 12/2026 | Chirag G. Patil | Merck Sharp & Dohme LLC\|National Cancer Institute (NCI) |
| NCT01269853 | Repeated Super-selective Intraarterial Cerebral Infusion of Bevacizumab (Avastin) for Treatment of Relapsed GBM and AA | Phase 1 \|Phase 2 | Recruiting | Bevacizumab | 10/2010 | 10/2026 | Northwell Health | Feinstein Institute for Medical Research |
| NCT03277638 | Laser Interstitial Thermotherapy (LITT) Combined With Checkpoint Inhibitor for Recurrent GBM (RGBM) | Phase 1 \|Phase 2 | Recruiting | Pembrolizumab at 7 days prior \| Pembrolizumab at 14 days post \| Pembrolizumab at 35 days post \| Laser Interstitial Thermotherapy | 11/2017 | 06/2025 | Case Comprehensive Cancer Center |  |
| NCT04085887 | Panitumumab-IRDye800 to Detect Pediatric Neoplasms During Neurosurgical Procedures | Phase 1 \|Phase 2 | Not Yet Recruiting | Panitumumab-IRDye800 \| Pinpoint-IR9000 endoscopic/handheld device \| Explorer Air camera \| PDE-NEO-II | 04/2024 | 01/2025 | Stanford University |  |
| NCT06047379 | Safety and Efficacy of NEO212 in Patients With Astrocytoma IDH-mutant, Glioblastoma IDH-wildtype or Brain Metastasis | Phase 1 \|Phase 2 | Recruiting | NEO212 Oral Capsule \| Ipilimumab \| Pembrolizumab \| Nivolumab \| Regorafenib \| Carboplatin \| Paclitaxel \| FOLFIRI Protocol \| Bevacizumab | 11/2023 | 08/2026 | Neonc Technologies, Inc. |  |
| NCT03603405 | HSV-tk and XRT and Chemotherapy for Newly Diagnosed GBM | Phase 1 \|Phase 2 | Recruiting | ADV/HSV-tk (gene therapy) | 02/2018 | 12/2025 | The Methodist Hospital Research Institute |  |
| NCT03596086 | HSV-tk + Valacyclovir + SBRT + Chemotherapy for Recurrent GBM | Phase 1 \|Phase 2 | Recruiting | ADV/HSV-tk (gene therapy) | 12/2017 | 12/2025 | David Baskin MD | Center for Cell and Gene Therapy, Baylor College of Medicine |
| NCT05084430 | Study of Pembrolizumab and M032 (NSC 733972) | Phase 1 \|Phase 2 | Recruiting | M032 \| Pembrolizumab | 02/2022 | 03/2028 | University of Alabama at Birmingham |  |
| NCT05096481 | PEP-CMV Vaccine Targeting CMV Antigen to Treat Newly Diagnosed Pediatric HGG and DIPG and Recurrent Medulloblastoma | Phase 2 | Recruiting | PEP-CMV \| Temozolomide \| Tetanus Diphtheria Vaccine | 07/2024 | 06/2030 | Nationwide Children's Hospital |  |
| NCT06264388 | DB107-Retroviral Replicating Vector (RRV) Combined With DB107-Flucytosine (FC) in Patients With Recurrent Glioblastoma or Anaplastic Astrocytoma | Phase 2 | Recruiting | DB107-RRV \| DB107-FC | 05/2024 | 05/2034 | Ashish Shah | Denovo Biopharma LLC\|National Cancer Institute (NCI) |
| NCT02691923 | Diagnostic Performance of Fluorescein as an Intraoperative Brain Tumor Biomarker | Phase 2 | Recruiting | Fluorescein \| Fluorescein + ALA | 03/2016 | 12/2026 | David W. Roberts | National Institute of Neurological Disorders and Stroke (NINDS)\|Carl Zeiss Meditec, Inc. |
| NCT06058988 | Trastuzumab Deruxtecan (T-DXd) for People With Brain Cancer | Phase 2 | Recruiting | Trastuzumab deruxtecan | 09/2023 | 09/2027 | Memorial Sloan Kettering Cancer Center | AstraZeneca |
| NCT06132685 | Post-Operative Dosing of Dexamethasone in Patients With Brain Tumors After a Craniotomy, PODS Trial | Phase 2 | Not Yet Recruiting | Biospecimen Collection \| Computed Tomography \| Dexamethasone \| Magnetic Resonance Imaging \| Questionnaire Administration | 10/2024 | 07/2028 | Emory University | National Cancer Institute (NCI) |
| NCT02800486 | Super Selective Intra-arterial Repeated Infusion of Cetuximab (Erbitux) With Reirradiation for Treatment of Relapsed/Refractory GBM, AA, and AOA | Phase 2 | Recruiting | Intra-arterial Cetuximab \| Intra-arterial Mannitol \| Hypofractionated re-irradiation | 05/2016 | 05/2026 | Northwell Health |  |
| NCT05864534 | Phase 2a Immune Modulation With Ultrasound for Newly Diagnosed Glioblastoma | Phase 2 | Recruiting | Balstilimab \| Botensilimab \| Liposomal Doxorubicin \| Sonocloud-9 (SC-9) | 01/2024 | 08/2026 | Northwestern University | Agenus Inc.\|CarThera |
| NCT02177578 | Subventricular Zone (SVZ) and Temozolomide in Glioblastoma Multiforme* | Phase 2 | Recruiting | Temozolomide \| Subventricular Zone radiation \| Neural Progenitor Cell Sparing radiation | 07/2014 | 12/2032 | Sidney Kimmel Comprehensive Cancer Center at Johns Hopkins | Reading Health System Foundation |
| NCT06061809 | N-803 and PD-L1 t-haNK Combined With Bevacizumab for Recurrent or Progressive Glioblastoma | Phase 2 | Recruiting | Bevacizumab \| PD-L1 t-haNK \| N-803 | 08/2024 | 12/2030 | ImmunityBio, Inc. |  |
| NCT05463848 | Surgical Pembro +/- Olaparib w TMZ for rGBM* | Phase 2 | Recruiting | Pembrolizumab \| Olaparib \| Temozolomide | 10/2022 | 12/2025 | L. Nicolas Gonzalez Castro, MD, PhD | Merck Sharp & Dohme LLC |
| NCT04222062 | A Study Comparing GLIADEL to Stereotactic Radiosurgery in Metastatic Brain Disease | Phase 2 | Recruiting | Carmustine 7.7Mg Wafer | 11/2020 | 12/2026 | University of Nebraska | Arbor Pharmaceuticals, Inc. |
| NCT06325683 | Anti-Lag-3 (Relatlimab) and Anti-PD-1 Blockade (Nivolumab) Versus Standard of Care (Lomustine) for the Treatment of Patients With Recurrent Glioblastoma | Phase 2 | Recruiting | Biopsy \| Biospecimen Collection \| Lomustine \| Magnetic Resonance Imaging \| Nivolumab \| Relatlimab \| Surgical Procedure | 05/2025 | 07/2028 | National Cancer Institute (NCI) |  |
| NCT05879250 | WP1066 and Radiation Therapy in Treating Patients With Newly Diagnosed Glioblastoma | Phase 2 | Recruiting | Biospecimen Collection \| Magnetic Resonance Imaging \| Radiation Therapy \| STAT3 Inhibitor WP1066 \| Surgical Procedure | 05/2024 | 12/2028 | Northwestern University | Moleculin Biotech, Inc.\|National Cancer Institute (NCI) |
| NCT04690348 | Intracavitary Carrier-embedded Cs131 Brachytherapy for Recurrent Brain Metastases: a Randomized Phase II Study | Phase 2 | Recruiting | Craniotomy \| Cesium-131 brachytherapy | 12/2020 | 12/2025 | Memorial Sloan Kettering Cancer Center | GT Medical Technologies, Inc. |
| NCT05305365 | Study Assessing QBS72S For Treating Brain Metastases | Phase 2 | Recruiting | QBS72S | 08/2022 | 08/2026 | Melanie Hayden Gephart | Quadriga Biosciences, Inc. |
| NCT04743310 | Fluorescence Detection of Adult Primary Central Nervous System Tumors With Tozuleristide and the Canvas System | Phase 2 | Recruiting | tozuleristide \| Canvas imaging system \| Surgical resection of tumor | 09/2021 | 02/2025 | John Yu | Blaze Bioscience Inc. |
| NCT04348747 | Dendritic Cell Vaccines Against Her2/Her3 and Pembrolizumab for the Treatment of Brain Metastasis From Triple Negative Breast Cancer or HER2+ Breast Cancer | Phase 2 | Recruiting | Anti-HER2/HER3 Dendritic Cell Vaccine \| Pembrolizumab | 12/2022 | 12/2026 | Roswell Park Cancer Institute | United States Department of Defense |
| NCT04339751 | Effect of Vorinostat on ACTH Producing Pituitary Adenomas in Cushing s Disease | Phase 2 | Recruiting | Vorinostat | 10/2024 | 12/2024 | National Institute of Neurological Disorders and Stroke (NINDS) |  |

Supplementary Table 3. Neurosurgeon-led phase 2 trials.
