# Supplementary material for: Neurosurgical leadership in neuro-oncology clinical trials: A nationwide study
Source: Neurosurg Rev. 2026 Mar 9;49(1):265. doi: 10.1007/s10143-026-04165-5 (PMC12971842; doi:10.1007/s10143-026-04165-5)
Supplement: Supplementary file 6 — Supplementary Material 5 (DOCX 24.8 KB) [file 10143_2026_4165_MOESM5_ESM.docx]

| **NCT Number** | **Study Title** | **Phases** | **Study Status** | **Interventions** | **Start Date** | **Completion Date** | **Sponsor** | **Collaborators** |
| --- | --- | --- | --- | --- | --- | --- | --- | --- |
| NCT05271240 | Repeated Superselective Intraarterial Cerebral Infusion (SIACI) of Bevacizumab With Temozolomide and Radiation Compared to Temozolomide and Radiation Alone in Newly Diagnosed GBM | Phase 3 | Recruiting | Repeated Superselective Intraarterial Cerebral infusion (SIACI) of Bevacizumab (Avastin) with Temozolomide and Radiation \| Temozolomide and Radiation Alone | 04/2022 | 04/2028 | Northwell Health |  |
| NCT06388733 | A Study Comparing Niraparib With Temozolomide in Adult Participants With Newly-diagnosed, MGMT Unmethylated Glioblastoma | Phase 3 | Recruiting | Niraparib \| Temozolomide | 06/2024 | 03/2028 | Ivy Brain Tumor Center | GlaxoSmithKline |
| NCT05317858 | Blood-brain Barrier (BBB) Disruption Using Exablate Focused Ultrasound With Standard of Care Treatment of NSCLC Brain Mets | Phase 3 | Recruiting | Blood Brain Barrier Disruption - Oncology \| Pembrolizumab | 08/2022 | 12/2024 | InSightec |  |
| NCT06448286 | PH Weighted Chemical Exchange Saturation Transfer MRI-Based Surgical Resection to Improve Survival in Patients With Glioblastoma | Phase 3 | Not Yet Recruiting | Chemical Exchange Saturation Transfer Magnetic Resonance Imaging \| Contrast-enhanced Magnetic Resonance Imaging \| Magnetic Resonance Imaging \| Radiation Therapy \| Surgical Procedure \| Temozolomide | 12/2024 | 12/2027 | Jonsson Comprehensive Cancer Center |  |
| NCT03750227* | Pre-Operative or Post-Operative Stereotactic Radiosurgery in Treating Patients With Operative Metastatic Brain Tumors | Phase 3 | Recruiting | Conventional Surgery \| Quality-of-Life Assessment \| Questionnaire Administration \| Stereotactic Radiosurgery | 11/2018 | 11/2025 | Mayo Clinic |  |
| NCT04365374 | Post-Surgical Stereotactic Radiotherapy (SRT) Versus GammaTile-ROADS (Radiation One and Done Study) | Phase 3 | Recruiting | Gamma Tile-Surgically Targeted Radiation Therapy (STaRT) \| Stereotactic Radiation Therapy | 04/2021 | 12/2027 | GT Medical Technologies, Inc. |  |
| NCT03014687 | Prophylactic Oral Antibiotics on Sinonasal Outcomes Following Endoscopic Transsphenoidal Surgery for Pituitary Lesions | Phase 4 | Recruiting | Placebo \| Oral Antibiotics cefdinir or trimethoprim/sulfamethoxazole | 07/2017 | 07/2019 | St. Joseph's Hospital and Medical Center, Phoenix | Thomas Jefferson University\|The Cleveland Clinic |

Supplementary Table 4. Neurosurgeon-led phase 3/4 trials.
